# Beyond the Checklist: Developing Effective Communication in a South African Multilingual Clinical Training Programme

**DOI:** 10.1007/s40670-026-02816-2

**Published:** 2026-07-04

**Authors:** DR Prakaschandra, L McNamee, G Hendricks

**Affiliations:** 1 Department of Biomedical and Clinical Technology, Faculty of Health Sciences, Durban University of Technology, Durban, South Africa; 2 Department of Health Sciences Education; Faculty of Health Sciences, University of Cape Town, Cape Town, South Africa

**Keywords:** Effective communication, Graduate attribute, Global South, Clinical Technology, South Africa, Health Sciences Education

## Abstract

**Introduction:**

Effective communication is a core graduate attribute (GA) in health professions education, yet its development is fragmented and under-theorised in under-resourced and multilingual contexts, which are typical of the Global South, where postcolonial histories and structural inequities continue to shape educational practice.

**Method:**

We investigated how effective communication is understood, experienced and developed in a South African Clinical Technology programme, using a qualitative design underpinned by Engeström’s Activity Theory as an interpretive lens. Interviews and focus groups were conducted with final-year students (*n* = 10), recent graduates (*n* = 5), academic educators (*n* = 4), and clinical educators (*n* = 6), and thematic analysis was applied.

**Results:**

Four themes emerged: (1) effective communication as a situated, adaptive practice; (2) Barriers to effective communication (linguistic, cultural, structural); (3) Role modelling and relational dynamics; and (4) Adaptability and resilience in workplace communication. Effective communication development was largely relational and experiential rather than shaped by formal curriculum, with students learning communication through immersion and mentorship amid systemic challenges. Structural misalignments between formal curricular goals and clinical realities constrained students’ communication practice and confidence.

**Conclusion:**

For educators, the findings suggest practical steps: 1) reconceptualise effective communication as a situated, socially mediated practice; (2) embed multilingual and multimodal communication opportunities into routine clinical teaching and assessment; (3) strengthen alignment and dialogue between universities and clinical training sites; and (4) prioritise faculty development to support inclusive role modelling and psychologically safe learning environments. Re-conceptualising effective communication as a situated, socially mediated practice (instead of a generic skill), enables intentional curriculum design and assessment that better prepare graduates for the complex socio-cultural clinical environments of the Global South.

## Introduction and Background

Effective communication (EC) is a core clinical skill and a key graduate attribute (GA) in health professions education [1], underpinning safe, ethical and collaborative clinical practice. Accreditation frameworks in South Africa explicitly position effective communication as a core graduate competency [2].Yet in multilingual and under-resourced contexts, typical across low-and-middle-income countries (LMICs), and the broader Global South (a relational term encompassing regions largely in Asia, Africa, and Latin America and marked by shared postcolonial and structural inequities) [3], effective communication development remains inconsistently reported and poorly theorised.

Studies from Nepal [4], Ghana [5], Malawi [6], Namibia [7] and South Africa [8] have documented persistent language discordance, fragmented institutional support and clinical learning environments which prioritise biomedical routines over culturally responsive communication [9].

Beyond academic success, effective communication is integral to professional identity formation and sustained clinical competence [10]. While the value of effective communication has been extensively documented in various healthcare disciplines, particularly through simulation, reflection and clinical workplace learning [11], existing research often assumes effective communication to be a universally defined and teachable generic, implicitly acquired set of skills [12]. However, this assumption overlooks the ways in which institutional context, linguistic diversity, and power dynamics among students, educators, and health systems in practice, shape communication in practice.

Most effective communication training models originate in high-income, English-dominant settings, and fail to address the layered complexities of multilingual healthcare environments [13] such as those prevalent in the Global South [14]. In these contexts, communication becomes a site of negotiation and adaptation [15], rather than a straightforward, transferable skill. Students are required to communicate across cultural, linguistic and professional boundaries in high-pressured environments, and often in English, which is not their first language. Recent work corroborates that foreign-language medical education can impair both learning and clinical communication, especially where clinical interaction occurs in patients’ home languages [16].

The context-specific challenges of developing effective communication in such environments are seldom examined, nor are they systematically scaffolded within curricula. Instead, these challenges are frequently framed as implementation failures rather than as evidence of conceptual misalignment between curricular intentions and clinical realities. Evidence from Latin America reinforces this point. A Delphi study across eight countries reached consensus on the importance of strong communication training in undergraduate medical education. However, institutional approaches varied widely in how communication was integrated and assessed, with regional standards often disconnected from clinical realities of practice [17].

These challenges are particularly pronounced in South Africa, where medical training occurs across linguistically diverse clinical settings, with limited and inconsistent support for communication in non–mother-tongue languages. Despite policy frameworks positioning effective communication as essential, many curricula reduce it to a decontextualised checklist, that fails to reflect the multilingual, under-resourced environments in which students train and later practice. Although frameworks such as AfriMEDS outline broad curriculum ideals [2, 18], these are inconsistently translated into coherent design or implementation across programmes. This disconnect highlights the need to examine how institutional curricular intentions align with the realities of healthcare education in the Global South.

Effective communication development is often inconsistently supported, especially in programmes lacking formal communication components [7, 19] and in settings shaped by linguistic and cultural diversity [7]. While communication is increasingly recognised as interwoven with issues surrounding language policy and epistemic access, students frequently lack structured preparation for the demands of a multilingual and multicultural clinical training environments. Many students report low confidence when using additional languages in clinical encounters [20], and language barriers continue to hinder effective care when structured institutional support is absent [21].

Clinical Technology (CT) exemplifies these challenges. As a health professions discipline focused on diagnostic and therapeutic procedures using advanced medical technology, CT requires both technical expertise and effective interpersonal communication, which in itself requires precision, adaptability and emotional strength. Despite its high-stakes nature, with training situated within critical care units, cardiac surgery theatres, and dialysis units, CT remains unexplored in educational research [22]. Moreover, while effective communication literature in health professions education has expanded, the focus is predominantly on nursing and medicine, and often adopts a deficit perspective, attributing communication challenges to individual shortcomings like limited language proficiency or confidence, while overlooking the structural, institutional or sociolinguistic factors.

This study is therefore well-positioned to address that gap and explored how effective communication is conceptualised, experienced, and developed within a South African CT programme, to inform curricular reform in comparable Global South contexts.

### Theoretical Framework

The development of effective communication as a graduate attribute is shaped by curriculum design, learning environments, institutional values, and the expectations of multiple stakeholders. Accordingly, to explore how these elements interact in our context we drew on Engeström’s Activity Theory [23], to examine perspectives of final-year students, recent graduates, academic and clinical educators, with a focus on the tensions between institutional goals and clinical realities. AT offers a sociocultural perspective on learning, emphasising that human activity is mediated by tools, rules, communities, and divisions of labour within a system [23]. From this perspective, learning is not simply the internalisation of knowledge or skills but the transformation of activity through participation in culturally and historically situated practices. In this study, effective communication is understood as a practice co-constructed through interactions among students, educators, clinical environments, and institutional structures.

AT is particularly suited to examining contradictions and tensions within educational systems, such as the misalignment between formal curricular goals and real-world clinical expectations. It provides a lens to investigate how students, academic and clinical educators experience the development of effective communication in different settings, and how these experiences are shaped by broader institutional and sociolinguistic factors. By mapping the components of the activity system, including subjects, tools (e.g., language, simulations), community (e.g., educators, peers, patients), rules (e.g., assessment practices), division of labour and outcomes (e.g., demonstration of effective communication in clinical practice), the study identifies points of convergence, dissonance, and aspects for potential transformation.

Using AT allowed us to surface the structural and cultural conditions that either enable or constrain effective communication development. It also helped clarify why stakeholders often hold different conceptualisations of effective communication and why these differences matter for educational practice.

## Methodology and Methods

This study employed a qualitative phenomenological design grounded in an interpretivist paradigm enabling an in-depth exploration of the lived experiences and meaning-making processes of students, new graduates, and educators in the Clinical Technology (CT) programme at a University of Technology. Phenomenology was selected to elicit rich, contextualised insights into how effective communication was experienced and enacted in clinical and academic settings, and because the study sought to understand how participants experienced and made sense of effective communication development within their training environments, rather than to measure predefined competencies [24].

### Participants and Setting

The study was conducted at a South African University of Technology, recognised as a previously disadvantaged institution from Apartheid. The CT programme serves a diverse student body, predominantly Black African and female, with a high proportion of first-generation university attendees. At the time of the study, 2601 students were enrolled in the Faculty of Health Sciences, including 180 in the CT undergraduate programme.

Participants were drawn from four stakeholder groups: final-year students (38 eligible), new graduates (25 eligible), four academic staff (4 eligible) and clinical educators (12 eligible).

### Sampling and Recruitment

Following ethical approval and gatekeeper permissions, purposive sampling was employed to identify participants with direct experience of effective communication development. Purposive sampling in this study refers to intentionally recruiting information-rich participants from each stakeholder group (students, graduates, academic educators, clinical educators) to capture variation in experiences across academic and clinical settings [25]. Sample adequacy was guided by the concept of information power rather than data saturation, recognising that in focused, theory informed qualitative studies, analytical depth and relevance to the research aim are more appropriate indicators of sufficiency than numerical redundancy, as articulated by Malterud, Siersma, and Guassora [26]. Participants were selected to provide in-depth, contextually grounded insights aligned with the study’s aims, rather than to achieve a specific quota.

Participants were eligible if they had completed or supervised clinical placements within the CT programme. Final-year students and new graduates were recruited through class announcements and email invitations. Academic and clinical educators were approached through official institutional communication channels. Participation was voluntary and written informed consent was obtained from all participants.

### Data Generation

Data were collected between June and September 2024, using four tailored instruments: two focus group discussion (FGD) guides and two interview guides, each adapted to the specific participant group. The guides were developed by the research team based on study aims and relevant literature and piloted with a small group of participants for each group, not included in the main study to refine clarity and sequencing. Using both FGDs and interviews enabled exploration of both collective and individual perspectives, enhancing data richness and supporting triangulation.

Three FGDs (two with students, one with new graduates) lasted approximately 60 min each. In addition, ten individual interviews were held with academic and clinical educators, ranging from 55 to 75 min in duration.

An independent interviewer, trained in qualitative methods, and familiar with Activity Theory, conducted the FGDs to minimise power dynamics; individual interviews were conducted by the first author, using the same semi-structured guide. All sessions were audio-recorded and transcribed verbatim. Analytical memos were maintained throughout data collection process and member checking was conducted through transcript verification to enhance credibility.

### Data Analysis

Data were analysed using Braun and Clarke’s six-phase thematic analysis [27, 28]. An inductive coding strategy was employed, allowing themes to emerge from participants’ narratives without imposing preconceived categories. NVivo (v 11) was used to manage data and support coding.

A preliminary codebook was developed comprising 92 descriptive codes (e.g. language barriers, role modelling, curricular gaps), which were iteratively refined and consolidated into 22 categories. These categories informed the development of four overarching themes. Consistent with the principle of information power, themes were retained where they demonstrated sufficient conceptual depth, explanatory value, and resonance across participants, even when expressed in varied ways.

While coding was inductive, interpretation was guided by Engeström’s Activity Theory (AT) [29], which provided a lens to examine systemic contradictions and mediating factors within the activity system. After initial coding, themes were mapped against AT components (subjects, tools, rules, community, division of labour, and outcomes), to explore how these elements shaped effective communication development.

To enhance rigour, two coders (first author and supervisor) independently reviewed all transcripts. Discrepancies were resolved through discussion and consensus. Reflexivity was maintained through memo-writing and supervisory audits throughout the analytical process.

### Ethical Considerations

This study was part of a larger investigation for a postgraduate degree. Ethical approval was granted by the Institutional’ s Research and Ethics Committee (HREC REF 317/2024).

Participation was voluntary, and individuals were informed of their right to withdraw at any time without consequences. To minimise potential distress, psychological support was made available to students who reflected on challenging or emotionally charged clinical experiences.

The lead researcher maintained a reflexive journal throughout the study and engaged in regular supervisory discussions to ensure analytic rigour and transparency.

Trustworthiness was established in accordance with Lincoln and Guba’s (1985) [30] criteria: Credibility through prolonged engagement, triangulation (multiple groups and data sources) and member checking; Dependability through a clear audit trail and the use of information power principles; Confirmability through collaborative coding and grounding in participants’ voices; and Transferability by providing thick descriptions of the context and participant experiences.

## Results

Ultimately, 10 (26%) students and 5 (20%) graduates participated, alongside 4 (100%) academic educators and 6 (50%) clinical educators. Final-year students and graduates reflected on their effective communication development across clinical placements. Academic educators provided insights into curriculum design and assessment, while clinical educators, who were based at affiliated training sites, offered perspectives into workplace-based learning and supervision practices. All clinical educators followed a standardised clinical curriculum.

Four central themes were identified: Effective communication as a situated, adaptive practice; Barriers to effective communication development (Linguistic, cultural and structural); Role Modelling and relational dynamics; and Adaptability and resilience in workplace communication. These themes illustrate how participants experienced the development of the effective communication graduate attribute (GA) within the CT programme.

Participants described effective communication as a situated, context-sensitive process shaped by clinical experiences, interpersonal relationships, institutional structures, and cultural complexity, instead of just as a static outcome. This reframing challenges dominant assumptions that effective communication can be universally taught or assessed, revealing it as a dynamic, relational, and negotiated practice, particularly within resource-constrained and multicultural contexts of the Global South. Although themes are presented separately for clarity, they are interrelated and highlight specific areas for pedagogical attention and institutional alignment. In practice, students’ experiences often reflected overlapping dynamics: for example, communication challenges were closely linked to power hierarchies and inconsistent or inadequate institutional support.

### Theme 1: Effective Communication as a Situated, Adaptive Practice

Participants across all the groupings consistently framed effective communication development as fluid and experiential, shaped through clinical immersion and evolving through formal and informal encounters. Students emphasised learning to communicate through high-pressure scenarios rather than structured teaching.

“We come across people with various backgrounds… that really helped me and shaped my communication skills.” (S2).

“It was daunting… presenting in front of doctors at Groote Schuur [tertiary hospital]. But that experience made me realise how much I’d grown.” (G1).

“My qualifieds [senior technologists] encouraged me to speak up… they understood that we are students and *were still* (sic) learning.” (S3).

“You’re working in a toxic environment… people screaming at you and embarrassing you.” (S8).

These accounts suggest that effective communication development often emerged through negotiated participation complex clinical scenarios, where students were required to adjust and refine their communication in response to unpredictable interpersonal and institutional dynamics. These findings suggest the need for structured opportunities to reflect on and debrief clinical communication experiences (e.g. post-handover reflection sessions).

### Theme 2: Barriers to Effective Communication Development (Linguistic, cultural and structural)

Participants described effective communication as more than the transmission of information; it involved navigating power, identity, and interpersonal boundaries. Language discordance and cultural norms were identified as major barriers. Students reported relying on gestures, translation apps, peer support, and memorised phrases to communicate with patients who did not speak English or isiZulu.

“The old grannies and grandfathers… they just don’t understand isiZulu or English.” (G2).

“We ask the sisters [qualified nursing staff] to teach us just those simple terms… so that we can communicate.” (G2).

Yet educators expressed concern about students’ responsiveness to subtle, non-verbal cues:

“Receptive communication… from patients’ body language… they are not always able to do this.” (C5).

Students also grappled with gendered and cultural norms, such as modesty during procedures or respecting spiritual items. *(These items were in the form of prayer beads*,* and religious artifacts like strings worn around the abdomen*,* which students were expected to handle with cultural sensitivity.)*

“She was very conservative… her husband wasn’t happy [about her removing her top].” (S2).

“I just take [the cultural rope] and give it to the parent… explain why we had to remove it.” (S4).

At times, cultural expectations clashed with workplace norms, and educators noted that gaps in cultural awareness could compromise patient care.

“We can’t talk back to elders… students won’t stamp their authority because of those cultural values.” (C1).

In addition to linguistic and cultural barriers, participants highlighted structural tensions. Academic educators cited heavy workloads and fragmented institutional support, while clinical educators noted limited dialogue between universities and clinical sites.

“Because of my workload, I’m unable to attend [communication development] workshops… the support is not there.” (A2).

“We’re very driven by it in Clinical Technology… but institutionally, it’s not really given the respect it needs.” (A1).

“There has to be dialogue… what clinicians demand of students, and how we prepare them.” (C6).

These findings highlight the need for systematic alignment between academic and clinical environments, and for institutional investment in supporting students and staff with communication training, that addresses both linguistic and cultural complexities.

#### Theme 3: Role Modelling and Relational Dynamics

Educators’ behaviours strongly influenced how students developed effective communication, particularly in relation to confidence and communication practices. Positive modelling encouraged student participation, while negative modelling contributed to feelings of exclusion.

“If staff can improve their communication, students will improve too.” (A2).

“It affects your confidence as a student… you feel like nothing you say is taken seriously.” (S3).

This highlights the importance of faculty development initiatives that promote inclusive, respectful and student-centred communication practices.

### Theme 4: Adaptability and Resilience in Workplace Communication

Participants described effective communication development as a form of contextual adaptability. Students were required to shift their communication styles to meet the demands diverse clinical settings, patient needs, and institutional expectations.

“You have to be more simplistic [uncomplicated] or [without medical terminology] or [free from jargon]… use examples so they can understand.” (S6).

“It’s overwhelming… some end up dropping out.” (G3).

“It was nerve-wracking… but I learned I need to speak up, or I put patients at risk.” (S3).

Educators supported this growth through mentoring structures such as buddy systems and clinical handovers, but noted that students sometimes lacked initiative or were excluded from meaningful participation in clinical practice.

“By third year, they start watching and learning… so they know how to handle it.” (C3).

“Two qualifieds [senior technologists] go into a mobile EEG… the student just sits there and misses out.” (C5).

These accounts suggest that effective communication is closely tied to students’ abilities to adapt to shifting clinical demands, manage emotional strain and navigate institutional hierarchies. Those who developed communicative resilience often did so through informal learning, self-reflection, and repeated exposure to challenging encounters. Others remained peripheral, particularly when opportunities for active participation were limited.

These findings reinforce the need for structured opportunities that support students’ communicative adaptability, for example, scaffolded clinical briefings, peer mentoring and explicit teaching of strategies for managing high-pressure encounters. Furthermore, effective communication assessment frameworks should include indicators of adaptability and resilience, rather than focussing solely on technical competence.

## Discussion

This study explored how the effective communication graduate attribute is developed in the Clinical Technology programme in South Africa, revealing effective communication as a dynamic, context-sensitive practice rather than a static, transferable skill. By examining perspectives across students, graduates, academic and clinical educators, the findings challenge the dominant assumptions that effective communication can be universally taught or assessed through generic frameworks. Instead, effective communication development emerged as a contingent, relational and shaped by overlapping linguistic, cultural, structural and interpersonal factors.

Our study contributes to a broader understanding of how graduate attributes such as effective communication can and cannot be meaningfully developed in multilingual, under-resourced contexts where teaching quality and learning support vary across programmes or settings. Furthermore, the findings offer actionable insights to inform curriculum development and faculty training by identifying how effective communication could be more intentionally embedded into teaching, assessment and supervision practices within clinical environments. Using sociocultural perspectives provided a more appropriate analytical lens in this postcolonial, multilingual context, that positions communication as a situated, relational practice. These findings therefore contribute to a growing but still limited body of literature from the global South on communication development in the health professions.

### Theme 1: Effective Communication as Situated, Adaptive Practice

Theme 1 emphasised that effective communication development is embedded in participants’ lived experiences rather than acquired through isolated teaching moments. Students developed through active participation in clinical environments where they were required to navigate ambiguous, high-pressure situations, communicate across professional hierarchies, and adapt to patient needs.

These findings align with Activity Theory and Sociocultural Theory, which posit that learning is mediated by instruments (e.g., handovers, and feedback), rules (institutional norms), and communities (clinical teams) [31]. Our study revealed fragmentation across these systems, requiring students to adapt to communication practices without explicit guidance. This resonates with prior work in South Africa, where health science students face racially and linguistically diverse health systems, with limited formal support for communication development [32].

The literature from the Global North often frames effective communication as a teachable set of techniques, such as interview skills, consultation frameworks, and teamwork, typically situated within a culturally homogenous, English-speaking setting [33]. While these approaches are valuable, they fall short of addressing the layered challenges of resource-constrained, multilingual, and culturally diverse environments [34]. Our findings support previous research [9, 35] that effective communication in LMIC and the Global South is under-supported. This interpretation supports the need for curriculum design that embeds effective communication development into authentic clinical experiences supported by structured reflection and culturally responsive pedagogy, rather than relying solely on generic activities like communication workshops.

### Theme 2: Barriers to Effective Communication Development: Linguistic, Cultural, and Structural

Theme 2 highlights the systemic and contextual barriers that compound effective communication challenges. Language discordance and cultural norms shaped students’ ability to communicate effectively, while structural misalignments such as fragmented responsibilities and limited dialogue between higher education institutions and clinical sites, exacerbated these difficulties. Activity Theory frames these tensions as contradictions between rules (policy frameworks) and tools (pedagogical strategies), and between the division of labour (academic vs. clinical educators) and the object of activity (developing effective communication). These contradictions disrupted students’ ability to participate fully in workplace learning [23].

Academic educators described curriculum documents that named effective communication but highlighted the few institutional supports or unified approaches for implementation. Clinical educators, meanwhile, emphasised high expectations for students to communicate effectively in dynamic settings, and pointed out the minimal scaffolding and consequent under preparedness of students to enter the clinical training spaces. Our findings resonate with a recent mixed-methods evaluation of AfriMEDS implementation in a South African undergraduate medical programme which highlighted that intrinsic competencies such as communication are frequently enacted at a superficial level, despite being prominently stated in national curriculum frameworks [36]. Educators reported limited preparation to teach and assess these roles, while assessment practices were often misaligned with AfriMEDS intent. These findings indicate that although effective communication was formally acknowledged, it was inconsistently supported, modelled and assessed in clinical training environments.

This misalignment is not unique to South Africa and is pronounced in the global South setting where institutions carry the legacy of inequality and underfunding [37]. Furthermore, first-generation students may lack the cultural capital and academic literacy, which may present more challenges when navigating professional norms [38]. Their development of effective communication is therefore a layered process that involves negotiation of legitimacy, identity, and belonging in contested spaces [39*)*.

These findings echo global literature on effective communication development in LMICs [9, 35] and highlights the need for institutional alignment and workload adjustments for educators to enable sustained communication training.

### Theme 3: Role Modelling and Relational Dynamics

Theme 3 demonstrates that effective communication development is shaped by relational dynamics and the quality of role modelling. Educators’ everyday communicative behaviours strongly influenced students’ confidence and emerging communication practices. Positive modelling invited participation, while negative modelling generated feelings of exclusion and silence. Therefore, educators’ modelling of professional dialogue, particularly in high-pressure settings, can either empower or silence students, with long-term implications for their confidence and clinical competence, and needs to be explicitly addressed by faculty development programmes.

These faculty development initiatives should prioritise inclusive, respectful and student-centred communication practices [39]. This interpretation aligns with sociocultural perspectives and supports calls for mentorship structures that model professional dialogue and enable students to speak up in hierarchical environments [40].

Role modelling remains central in health professions education for professional identity formation and communication competence in health professions education. Cruess and colleagues describe it as a powerful teaching strategy because it exposes students to professional behaviours in authentic contexts, allowing them to observe how clinicians and educators communicate with patients, families, and colleagues [41].

When educators model respectful, patient-centred and team-oriented communication, students tend to internalise and reproduce these norms in practice. In contrast, hierarchical cultures that silence junior voices can erode students’ sense of belonging and reduce their willingness to advocate for patient safety [9]. Evidence from systematic and empirical work indicates that faculty development and mentoring programmes that heighten educators’ awareness of their influence as role models, strengthen feedback practices, and create psychologically safe learning environments can improve students’ engagement, confidence, and professionalism [42].

In this study, students described continuously assessing the communicative climate to decide when and how they could speak. This shaped both their immediate effective communication behaviours and their longer-term sense of belonging in the CT community of practice. These findings reinforce the importance of mentorship structures that help students to find their voice within hierarchical clinical environments [43].

### Theme 4: Adaptability and Resilience in Workplace Communication

Theme 4 highlights effective communication development as a form of contextual adaptability and resilience in the clinical workplace. Participants described communication as relational, context-sensitive, and entwined with culture, power, and language. Students adapted their communication style across diverse clinical settings, simplified technical language for patients, and managed the emotional and cognitive demands of multitasking in high-pressure environments, often while negotiating linguistic and cultural differences [16, 32] .

In this context, values commonly emphasised in effective communication training, such as clarity, empathy, and structure, were interpreted through local cultural norms and hierarchies [16, 35]. For example, students raised in traditions of deference to elders struggled to assert themselves with senior clinicians or older patients, even when safety concerns were evident. Others described translating procedures and showing cultural sensitivity during clinical encounters [43].

Yet such adaptive, culturally responsive practices are seldom reflected in conventional effective communication rubrics, which tend to privilege standardised verbal performance over multilingual, multimodal negotiation [9, 19]. Within an Activity Theory lens, these experiences reveal tensions between rules (e.g. language expectations, assessment policies) and tools (e.g. informal peer support, improvised translation strategies), and between the division of labour (junior students versus senior clinicians) and the expectation that students should take initiative [23, 29]. These contradictions constrained students’ participation and contributed to persistent communication challenges.

This emphasis on adaptability and resilience aligns with emerging literature that positions these as essential graduate attributes in rapidly changing and resource-constrained healthcare systems [5, 25]. Resilience is understood as a process of managing, adapting and responding to significant stressors, through communication, reflection, and emotional regulation [44]. Our findings support this view: some students developed communicative resilience through self-reflection, informal mentoring, and repeated exposure to difficult encounters, learning when to speak up, how to de-escalate conflict, and how to maintain patient-centred communication despite pressure. Others remained peripheral, especially where hierarchical norms or limited opportunities reduced active participation [45, 46].

### Recommendations for Health Professions Education in the Global South

This study suggests several actionable recommendations for educators, curriculum designers, and HEIs.

First, effective communication should be reconceptualised as an embedded, adaptive practice; one that emerges through multilingual, multimodal and culturally responsive interaction. In settings where communication is influenced by cultural values, such as harmony, respect for hierarchy [47, 48] as well as diverse health beliefs [47], such as LMIC and the Global South contexts, effective communication cannot be reduced to generic, decontextualised skills training. For example, curricula can include local language phrase training, non-verbal communication workshops, and discussions about cultural humility.

Second, stronger alignment is needed between HEIs and clinical training sites to ensure a shared a common understanding of how effective communication is developed, assessed and supported. Regular forums or joint workshops can ensure that what clinical supervisors expect aligns with what universities teach. Both sides must recognise that students develop communication competence through observation, mentorship and informal learning (via non-verbal modes), as much as through formal instruction.

Third, academic and clinical educators need targeted support to model inclusive, respectful, and context-aware communication practices. Faculty development should explicitly address the impact of role modelling and equip educators to create psychologically safe, culturally attuned learning environments [49]. This may be training on constructive feedback, techniques to encourage student participation, and reflective activities on implicit bias and power dynamics. Further work is needed to examine how multimodal approaches, such as non-verbal and visual communication alongside speech, can strengthen empathy, clarity, and patient care.

Finally, consistent with Mnguni’s [2] critique of unevenly enacted curriculum frameworks we call for urgent curriculum reform that makes effective communication an explicit, assessable, and integrated GA across academic and clinical learning sites. In LMIC contexts, where linguistic, cultural, and systemic barriers persist, communication training must prepare students to become ethically grounded, socially responsive communicators, rather than solely technical competent [50]. Practically, this means embedding communication objectives and assessments into every clinical module, offering credit-bearing language support electives, and assessing students on teamwork and empathy alongside technical competence.

### Strengths and Limitations

This study offers several strengths. First, it addresses a significant gap in the literature in an under-researched CT programme. Furthermore, it foregrounds the perspectives of multiple stakeholders, providing a rich, multi-perspective understanding of effective communication development in a multilingual and under-resourced context. However, the study also has limitations: the sample was drawn from a single institution, which may limit generalisability of findings to other contexts. In addition, while the study focussed on multilingualism and cultural complexity, it did not include patients’ perspectives, which would have further enriched the understanding of communication dynamics in this clinical context.

Therefore, further research could explore effective communication development across multiple institutions and include patient perspectives to triangulate findings and deepen insights into communication practices in diverse clinical environments.

## Conclusion

By centring the lived experiences of CT students and educators at a South African university of technology, this study reframes effective communication as a socially mediated graduate attribute that develops through navigating complex, sometimes contradictory learning environments, shaped by systemic, cultural and relational dynamics.

These insights have relevance beyond South Africa. They offer guidance for curriculum reform in under-resourced, multilingual, and structurally diverse settings. Meaningful effective communication development requires moving beyond generic frameworks to engage with the social, institutional, and cultural conditions that shape communication in practice. Embedding effective communication into authentic clinical learning, supported by intentional pedagogy and inclusive role modelling, is essential to preparing graduates for the realities of healthcare in the Global South.
